# The Effectiveness of Photobiomodulation Therapy on Pain and Function in Patients with Patellofemoral Pain Syndrome—A Systematic Review and Meta-Analysis

**DOI:** 10.3390/jcm15010020

**Published:** 2025-12-19

**Authors:** Mohamed Salaheldien Alayat, Roaa A. Sroge, Abdulaziz Awali, Ammar Fadil, Omair Belal Malibari, Raad Hatim Ajawi, Eyad Noor Wali, Suhail Hafiz, Sameer Yamani

**Affiliations:** 1 Medical Rehabilitation Sciences Department, College of Applied Medical Sciences, Umm Al-Qura University, Mecca 21955, Saudi Arabia; rasroge@uqu.edu.sa (R.A.S.); amawali@uqu.edu.sa (A.A.); asfadil@uqu.edu.sa (A.F.); pt.omair.malibari@hotmail.com (O.B.M.); raadajawi@gmail.com (R.H.A.); eyadnoor.w@gmail.com (E.N.W.); suhailhaviz@gmail.com (S.H.); 2 Basic Science Department, Faculty of Physical Therapy, Cairo University, Giza 12613, Egypt; 3 Physical Therapy Department, Umm Al-Qura University Medical Center, Mecca 21955, Saudi Arabia; syamani@uqu.edu.sa

**Keywords:** photobiomodulation, patellofemoral pain syndrome, pain, function, systematic review, meta-analysis

## Abstract

**Objectives**: The aim of this systematic review was to evaluate the effectiveness of photobiomodulation (PBM) on pain and function in individuals with Patellofemoral Pain Syndrome (PFPS). **Methods**: A systematic review and meta-analysis were conducted in accordance with PRISMA guidelines. Search was performed across PubMed/Midline, Scopus, Web of Science, EBSCO, ScienceDirect, Wiley Online Library, Springer, Cochrane CENTRAL, PEDro, ResearchGate, and Google Scholar from inception to January 2025. Randomized controlled trials (RCT) examining PBM in individuals with PFPS were included. Data extraction, risk-of-bias assessment (RoB 2), and quality of evidence evaluation (GRADE) were performed independently by multiple reviewers. Primary and secondary outcomes were pain and function, respectively. A random effect meta-analysis was performed to estimate the standardized mean difference (SMD) at 95% confidence interval (CI) and overall effect size. **Results**: Eight trials (340 participants) met the inclusion criteria. PBM significantly reduced pain compared with the control (SMD = −0.83; 95% CI −1.40 to −0.27). Functional outcomes demonstrated a significant improvement favoring PBM (SMD = 0.68; 95% CI 0.08 to 1.27), although substantial heterogeneity was present (I^2^ = 83%). RoB2 showed five high-risk studies. GRADE showed a very low quality of evidence due to study limitations, imprecision, and inconsistency which limit the confidence to the effect estimate. **Conclusions**: PBM, combined with exercise, provides improvements in pain and knee function in individuals with PFPS. While findings support PBM as an effective adjunct modality, standardized dosing protocols and larger, high-quality RCTs are needed to strengthen future clinical recommendations.

## 1. Introduction

Patellofemoral Pain Syndrome (PFPS) is one of the most common knee disorders affecting adolescents and young adults, especially females and those who are physically active [1,2]. It is marked by pain around or behind the kneecap in the absence of identifiable intra-articular pathology. Epidemiological studies estimate that PFPS accounts for up to 7% of knee-related complaints in clinical settings, with a higher prevalence among female athletes [3,4]. While its exact etiology remains unclear, altered patellar tracking and impaired patellofemoral biomechanics are widely considered to be key contributors to symptom development [5,6]. Although PFPS is primarily a clinical diagnosis, imaging modalities such as X-ray, magnetic resonance imaging (MRI), and musculoskeletal ultrasound can support the assessment by identifying patellar malalignment, cartilage changes, and associated soft-tissue abnormalities when clinically indicated [7,8,9].

Clinically, individuals with PFPS typically report diffuse anterior knee pain that worsens with activities involving knee flexion, such as stair climbing, squatting, running, or prolonged sitting [5,6]. The condition is understood to be multifactorial, with contributing factors including biomechanical deficits, muscular imbalances, soft-tissue dysfunction, and repetitive overuse [10]. Altered patellar tracking and malalignment are central biomechanical features of PFPS and recent imaging studies have demonstrated that abnormalities in patellofemoral joint kinematics (such as lateral tilt, maltracking, and rotational instability) play a critical role in symptom persistence. Dynamic MRI investigations have confirmed that patellar tracking abnormalities increase patellofemoral joint stress and correlate with pain severity [11].

Structural factors such as medial patellofemoral ligament insufficiency and trochlear dysplasia further contribute to instability and aberrant patellar motion [12]. Surgical and biomechanical correction procedures, such as distal femoral osteotomy for valgus malalignment, have been shown to improve patellar stability and knee function, reinforcing the importance of alignment in PFPS pathophysiology [11]. Additionally, soft-tissue imbalances and altered neuromuscular activation patterns of the quadriceps may influence patellar tracking and exacerbate symptoms [13]. Without appropriate intervention, PFPS can result in persistent pain, quadriceps weakness, and long-term functional limitations that negatively impact quality of life [14,15]. Increased loading of the patellofemoral joint during flexion-based activities is believed to enhance nociceptive input, which helps explain the typical pattern of symptom aggravation.

A recent meta-analysis summarized the various therapeutic strategies used in managing PFPS [16]. Interventions such as knee-focused strengthening, hip muscle training, and postural stabilization exercises have shown positive effects on lower-limb biomechanics and symptom relief [17,18,19]. Additional treatment methods including taping, orthoses, bracing, biofeedback, and photobiomodulation (PBM) have been investigated as adjuncts to conventional rehabilitation programs [5,20,21,22,23,24].

PBM, previously referred to as low-level laser therapy, diode cluster therapy, high-power or high-intensity laser therapy, is a non-invasive approach that utilizes specific light wavelengths to stimulate cellular responses and facilitate tissue repair [25]. PBM has been extensively studied in musculoskeletal disorders such as knee osteoarthritis and rheumatoid arthritis, with findings suggesting notable reductions in pain and improvements in function [26,27,28,29,30,31].

The proposed mechanisms of PBM include enhanced mitochondrial adenosine triphosphate (ATP) production, modulation of inflammatory mediators, increased microcirculation, and activation of cellular repair pathways [32]. In the rehabilitation context, PBM has demonstrated potential benefits across a range of conditions, including tendinopathies, osteoarthritis, and muscle fatigue [32]. Clinical efficacy appears to be dose-dependent, with therapeutic outcomes commonly associated with wavelengths in the 810–1064 nm range and energy densities between 4 and 12 J/cm^2^.

The biological justification for PBM in treating PFPS refers to its analgesic, anti-inflammatory, and regenerative effects, which are believed to be mediated through mitochondrial cytochrome-c oxidase activation and subsequent modulation of cellular metabolism [33,34,35]. These physiological processes may result in clinically significant reductions in pain and functional impairment, as seen in other musculoskeletal conditions where PBM has yielded promising outcomes [36,37,38].

Considering the substantial burden of PFPS and ongoing uncertainty regarding optimal conservative treatment, it is important to assess novel interventions like PBM using rigorous and systematic methods. Clarifying the therapeutic value of PBM for PFPS can support evidence-based practice, help determine ideal dosing protocols, and guide clinical decision-making. The primary objective of this systematic review and meta-analysis was to evaluate the effectiveness of PBM in reducing pain and improving function in individuals with PFPS. The secondary objective was to investigate whether PBM, when used alone or alongside other physical therapy interventions, may provide superior therapeutic benefits.

## 2. Materials and Methods

### 2.1. Study Design

This review was conducted as a systematic review and meta-analysis in accordance with the Preferred Reporting Items for Systematic Review and Meta-Analysis (PRISMA 2020) guidelines [39,40] (Supplementary Material). The study protocol was prospectively registered in the PROSPERO International Prospective Register of Systematic Reviews (Registration ID: CRD420251185393). All methodological procedures followed the recommendations outlined in the Cochrane Handbook for Systematic Reviews of Interventions [41], ensuring transparent, reproducible, and methodologically rigorous evaluation of the available evidence.

### 2.2. Search Strategy

A comprehensive and systematic literature search was undertaken to identify all relevant studies evaluating the effects of PBM on PFPS. The search process was structured according to the Population, Intervention, Comparison, and Outcome (PICO) framework. Multiple electronic databases were searched from their inception to the most recent search date, including PubMed/MEDLINE, Scopus, Web of Science, CINAHL (EBSCOhost), Wiley Online Library, ScienceDirect, SpringerLink, the Cochrane Central Register of Controlled Trials (CENTRAL), and the Physiotherapy Evidence Database (PEDro). To ensure comprehensive coverage, additional sources such as Google Scholar and ResearchGate were screened for gray literature, conference proceedings, ongoing studies, and unpublished trials.

The search strategy combined controlled vocabulary terms and Medical Subject Heading (MeSH) and with free-text keywords related to the condition, intervention, and study design. Key terms included: “patellofemoral pain syndrome”, “anterior knee pain”, “chondromalacia patellae”, and “patellofemoral dysfunction” paired with “photobiomodulation therapy”, “low-level laser therapy”, “high-intensity laser therapy”, “laser therapy”, “light therapy”, “PBM”,”HPLT”, “LLLT”, and “HILT”, combined with filters such as “randomized controlled trial”, “clinical trial”, and related synonyms. Reference lists of eligible studies and relevant systematic reviews were manually screened to identify additional publications. Searches were performed from database inception to the final updated search. Search was limited to studies published in English, involving human participants, and using RCT or clinical trial.

All search activities, including databases searched, search terms used, dates of last search, and the number of retrieved results, were documented. The study selection process was presented using a PRISMA 2020 flow diagram, detailing the number of records identified, screened, excluded, and included in the final review.

### 2.3. Eligibility Criteria

Studies were considered eligible if they met the following criteria: (1) Population: Included participants diagnosed with PFPS, irrespective of sex, ethnicity, or activity level. (2) Study Design: RCTs or controlled clinical trials investigating the therapeutic effects of PBM. (3) Outcomes: reported at least the primary or secondary outcomes (pain intensity or functional performance). (4) Language: published in English.

Studies were excluded due to the following: (1) The participants presented with other knee pathologies, such as meniscal injuries, ligamentous or tendinous lesions, knee osteoarthritis, patellar instability, or any postoperative conditions. (2) The article was published in a non-English language. (3) PBM was delivered as part of the control or comparator intervention, preventing the isolation of its therapeutic effects (4). Pain or functional outcomes were not reported among the measured variables.

### 2.4. Study Selection

Two independent reviewers (OM and RA) screened all retrieved records to determine their eligibility based on the predefined inclusion and exclusion criteria. All references identified through the database search were imported into EndNote (version X9) to facilitate citation management and automatic duplicate removal.

During the first screening phase, titles and abstracts were evaluated and studies clearly unrelated to PBM or PFPS were excluded. Full-text versions of potentially eligible studies were then obtained and independently assessed by the same reviewers. Any disagreements regarding study eligibility were resolved through discussion and, when necessary, by consultation with a third senior reviewer (AW).

### 2.5. Data Extraction Process

Data extraction was independently performed by two reviewers (EW and SH) using a standardized extraction form developed specifically for this review. For each included study, the following information was collected: author(s), year of publication, study design, participant characteristics, intervention and comparator details, outcome measures, and key findings. A supplementary extraction sheet was used to document detailed PBM parameters, including PBM type, wavelength, frequency, power output, fluence (energy density), total delivered energy, treatment frequency per week, total number of sessions, and overall treatment duration. Extracted information was cross-checked between the two reviewers to ensure accuracy and consistency. In instances of incomplete information or discrepancies, a third reviewer (AF) was consulted to achieve consensus.

### 2.6. Risk-of-Bias Assessment

The methodological quality of all included RCT were independently evaluated by two reviewers (RS and AF) using the Cochrane Risk of Bias 2.0 (RoB 2) tool, in accordance with the guidelines outlined in the Cochrane Handbook for Systematic Reviews of Interventions [41]. An Excel template was used to document risk-of-bias judgments, supporting justifications, and direct quotations from the original studies. Any disagreements between reviewers were resolved through discussion and, when necessary, by consulting a third senior reviewer (MA).

RoB 2 assessed the bias across five domains: (1) bias arising from the randomization process, (2) bias due to deviations from intended interventions, (3) bias due to missing outcome data, (4) bias in measurement of the outcome, (5) bias in selection of the reported result. Each domain was assigned a rating of “low risk,” “some concerns,” or “high risk.” These judgments were used to generate an overall RoB rating for each included study. Domain-level and overall assessments were compiled into a summary table and a graphical visualization was produced.

### 2.7. Quality of Evidence Evaluation

The quality of evidence was evaluated using the Grading of Recommendations, Assessment, Development, and Evaluation (GRADE) framework. The assessment was conducted separately for pain intensity (measured using the Visual Analog Scale (VAS) or Numeric Pain Rating Scale (NPRS), 0–10 cm) and functional outcomes (measured using the Kujala Anterior Knee Pain (AKPS Scale)). Evidence quality was rated as high, moderate, low, or very low after considering the five standard GRADE domains: risk of bias, inconsistency, indirectness, imprecision, and publication bias [42].

Studies were grouped based on comparable intervention types (e.g., PBM alone versus control, or PBM combined with exercise versus control) to ensure meaningful synthesis. As RCTs represent the highest level of study design, the initial certainty rating for each outcome began as high. Downgrading occurred when concerns were identified in any GRADE domain.

For risk of bias, judgments were informed by the RoB 2 assessments across studies contributing to each pooled comparison. For inconsistency, evidence was downgraded when substantial heterogeneity was present (e.g., high I^2^ values, wide or non-overlapping confidence intervals), indicating variability in treatment effects across studies. For imprecision assessment, a minimally contextualized approach in line with updated GRADE guidance was used. Imprecision was judged relative to established minimally important differences (MIDs). For pain, the MID was defined as 1.5–2.0 points on a 0–10 VAS or NPRS [43]. For AKPS functional outcomes, the MID was set at 8–10 points on the 0–100 scale [43]. Rating down occurred when confidence intervals crossed MID thresholds or when sample sizes were insufficient.

For indirectness, judgments were downgraded when outcome measures, populations, or interventions deviated from the review question (e.g., different function scales, mixed patient populations). For publication bias assessment, visual inspection of funnel plots was used when ≥10 studies were available for a given outcome. A Summary of Findings (SoF) table was generated to present the pooled effect estimates and the corresponding GRADE certainty ratings for each outcome, along with explanations for any downgrading decisions.

### 2.8. Data Analysis

Meta-analyses were conducted using Review Manager 5.4 (RevMan) for Windows to calculate pooled effect sizes. Effect sizes were interpreted according to Cohen’s criteria, where 0.2 indicates a small effect, 0.5 a medium effect, and 0.8 or greater a large effect [44]. For pain intensity outcomes, standardized mean differences (SMD) with 95% confidence intervals (CI) were calculated to account for variations in pain scales across studies. Statistical heterogeneity was assessed using the I^2^ statistic, with thresholds of 0–40% indicating low heterogeneity, 30–60% moderate, and 50–90% substantial heterogeneity [45].

Functional performance was evaluated using either AKPS, where higher scores denote better knee function, or by the WOMAC, where higher scores indicate greater functional limitation. To harmonize the direction of effects between these scales, WOMAC scores were multiplied by −1 so that all functional measures reflected improvement with higher scores. Forest plots were generated to visually present pooled SMDs and their corresponding 95% confidence intervals.

Sensitivity analyses were conducted to assess the robustness of the findings by excluding studies judged to be at high risk of bias and those using non-standard PBM treatment parameters. Subgroup analyses (e.g., PBM alone versus PBM combined with exercise) were performed to explore potential sources of heterogeneity and to identify clinically meaningful effect modifiers. Where credible subgroup effects were detected, certainty of evidence was rated separately for the corresponding subgroups.

## 3. Results

### 3.1. Study Selection

A total of 1715 records were identified through comprehensive database searching. The number of studies was obtained from published articles in databases (1518) and non-databases such as ResearchGate, Google Scholar, and Citation searching (197). After removal of ineligible studies, 171 articles were considered potentially relevant based on title and keyword matching prior to screening.

**Figure 1:**
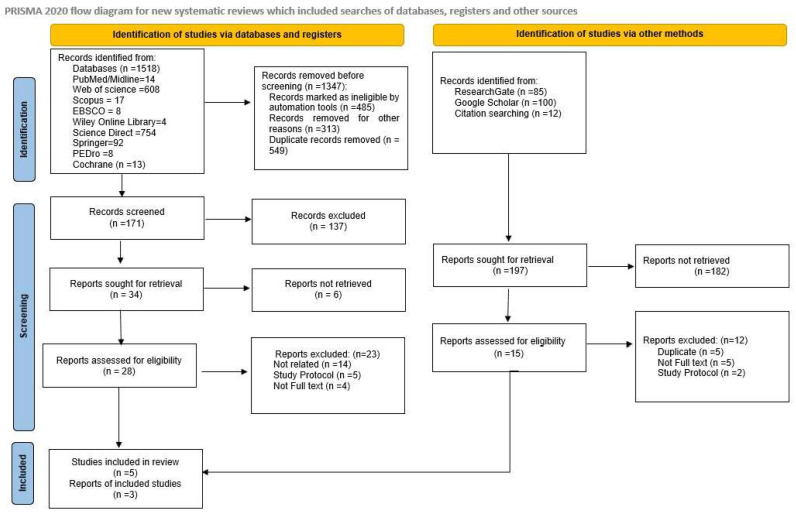
Flow diagram of the study selection process.

Following title and abstract screening, 28 articles from databases and 15 articles from websites met the eligibility criteria for full-text review. These studies were assessed in detail to determine their relevance to PBM interventions for PFBS. After applying the predefined inclusion and exclusion criteria, eight studies fulfilled all requirements and were included in the final qualitative synthesis and quantitative meta-analysis [5,23,24,46,47,48,49,50]. The remaining full-text articles were excluded due to reasons such as non-randomized study design, study protocol, inappropriate comparator groups, non-PBM interventions, or absence of pain/function outcome measures (Figure 1).

### 3.2. Study Characteristics

Eight single- or double-blind RCT published between 2019 and 2025 met the eligibility criteria and were included in the final review [5,23,24,46,47,48,49,50]. All studies evaluated the effectiveness of PBM in individuals diagnosed with PFPS [5,24,46,47,49,50], Anterior knee pain [23], or Bilateral patellofemoral OA [48] with an age range of 15–50 years (Table 1). Sample sizes across studies ranged from 18 to 60 participants, with most trials including both male and female participants—except one study on females only [49]—aged between late adolescence and early adulthood. All studies used clinically recognized diagnostic criteria for PFPS (Table 1).

All included studies evaluated pain as a primary outcome using VAS [5,23,24,46,47,49,50] or NPRS [48]. Functional performance was typically assessed using the AKPS [5,23,24,46,47,49,50] or using Western Ontario and McMaster Universities Osteoarthritis Index (WOMAC) [24,47,48], with some studies incorporating additional measures such as range of motion [46] or performance-based tasks [5] (Table 1). In AKPS, higher scores indicate better knee function and fewer symptoms, while in WOMAC, higher scores indicate worse pain, stiffness, and greater functional limitations.

The included trials assessed PBM either as a standalone treatment [49] or in combination with exercise therapy or physiotherapy programs [5,23,24,46,47,48,50]. Comparators varied and included sham laser, placebo PBM, conventional exercise programs, electrotherapy modalities like Transcutaneous Electrical Nerve Stimulation (TENS), ultrasound (US), interferential current (IFC) [5], or standard physiotherapy [23,50] (Table 1).

Across eight RCTs, all studies measured pain using VAS [5,23,24,46,47,49,50] or NPRS [48]. PBM showed favorable effects on pain and function in individuals with PFPS. Four studies reported significant pain reduction following PBM compared to sham or standard treatments [23,24,46,50], with Allam et al. 2025 showing the most notable improvement in VAS scores [46]. Studies combining PBM with exercise showed additional benefits over exercise alone, especially at long-term follow-ups for both pain and AKPS scores [5,24,48]. On the other side, Pocai et al. 2020, using a cluster PBM device in young women, found limited pain relief overall but reported functional gains in tests like jump landing [49]. Moreover, Eurcherdkul et al. 2023 observed no significant difference between high-intensity laser plus exercise and exercise alone, though both groups improved over time [47] (Table 2).

Functional outcomes, often assessed with the Kujala AKPS, improved in several studies [5,46] while others reported only modest or no changes. Despite variability in PBM protocols and study designs, the collective evidence supports PBM, particularly when paired with structured exercise, as a potential contributor to pain reduction and functional improvement for PFPS patients.

### 3.3. Evaluation of Methodological Quality

Methodological quality across the included trials ranged from low to high risk of bias, with variability primarily related to randomization processes, blinding procedures, and transparency in reporting. Three studies were assessed as low risk, supported by clear randomization, effective allocation concealment, and credible double-blinding using sham devices [23,46,47]. Five studies were judged to have two or more of some concerns [24,48,49,50] or one high-risk domain in the five evaluating domains and were considered as high risk-of-bias studies [5].

**Figure 2:**
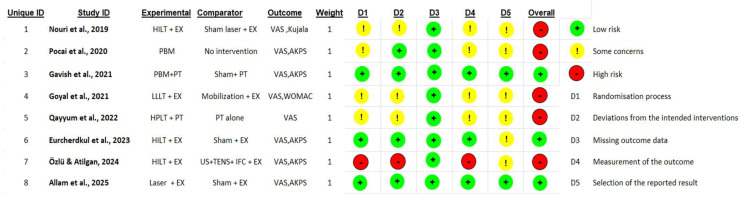
Traffic-light visualization of RoB 2 assessment of included studies [5,23,24,46,47,48,49,50].

The most common methodological risks involved allocation concealment, lack of therapist blinding, and unclear pre-specification of statistical analyses. In contrast, missing outcome data was generally well handled, with most trials achieving complete follow-up (Figure 2).

### 3.4. GRADE

For pain intensity, all eight included studies (340 participants) assessed pain using the VAS or NPRS on a 0–10 scale [5,23,24,46,47,48,49,50]. The certainty of evidence for pain was downgraded by three levels for study limitations, inconsistency, and imprecision with a very low overall quality of evidence. The pooled effect demonstrated a high effect size reduction in pain (SMD = −0.83; 95% CI −1.40 to −0.27), favoring PBM.

Function was measured by the AKPS [5,23,24,46,49] or WOMAC [24,47,48] scales. All included RCT studies (314 participants), except Qayyum et al., 2022 [50], measured the functional level and contributed data to this analysis. The pooled effect demonstrated a moderate improvement in function (SMD = 0.68; 95% CI 0.08 to 1.27), favoring PBM. The certainty of evidence for function was downgraded by three levels for study limitations, inconsistency, and imprecision and was rated as very low.

Study limitations were downgraded because several trials had unclear allocation concealment, inadequate blinding of participants, assessors, or therapists and five studies were judged to have high risk of bias. Inconsistency in pain and function outcomes were downgraded, owing to substantial statistical heterogeneity across trials (I^2^ > 75%), reflecting variation in PBM parameters, treatment combinations, and comparator groups.

Imprecision was rated down because the pooled 95% confidence interval crossed the MID threshold, indicating that the true effect may not reach a clinically meaningful reduction in pain for all patients. In addition, several included trials had small sample sizes, further widening the confidence interval and limiting certainty in the estimate. Although the pooled SMD indicated a large statistical reduction in pain, the imprecision reduces confidence that this effect consistently meets the established MID across studies.

For functional outcomes, imprecision was also rated down because the confidence interval of the pooled SMD crossed the established MID for both AKPS and WOMAC functionality scores. This suggests uncertainty regarding whether PBM yields a clinically meaningful improvement in function. Small sample sizes in some trials additionally contributed to imprecision. Thus, although the pooled estimate favored PBM with a moderate effect size, the true magnitude of improvement may be smaller and may not consistently reach clinical relevance (Table 3).

### 3.5. Meta-Analysis

#### 3.5.1. Effect of PBMT on Pain

The pooled analysis using a random-effects model demonstrated that photobiomodulation therapy (PBM) produced a statistically significant reduction in pain compared with control interventions (SMD = −0.83; 95% CI −1.40 to −0.27). Although the direction of the effect consistently favored PBM across studies, the between-study heterogeneity was substantial (I^2^ = 83%), suggesting notable variability in treatment responses.

**Figure 3:**
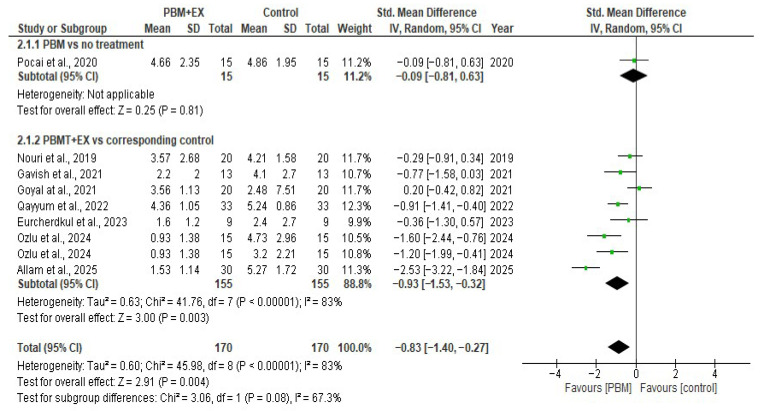
Forest plot of effectiveness of PBM on pain modulation [5,23,24,46,47,48,49,50].

Subgroup analysis comparing PBM combined with exercise versus corresponding controls also demonstrated a significant reduction in pain (SMD = −0.93, 95% CI −1.53 to −0.32, *p* = 0.003), though heterogeneity remained high (I^2^ = 83%). A single study comparing PBM alone with no treatment showed a non-significant reduction in pain (SMD = −0.09, 95% CI −0.81 to −0.63; *p* = 0.81). Overall, the findings suggest that PBM reduces pain intensity in individuals with PFPS, although the magnitude of benefit varies between studies, and methodological differences contribute to the high degree of heterogeneity (Figure 3).

#### 3.5.2. Effect of PBMT on Function

Seven comparisons from randomized controlled trials (314 participants) reported functional outcomes using either the AKPS or WOMAC scales. The pooled analysis using a random-effects model demonstrated a moderate, statistically significant improvement in function following PBM compared with control interventions (SMD = 0.68; 95% CI 0.08 to 1.27; *p* < 0.00001). Statistical heterogeneity was high (I^2^ = 83%), likely reflecting differences in PBM parameters (wavelength, energy dose, mode of delivery), treatment duration, co-interventions, functional measurement scales, and variations in comparator groups.

**Figure 4:**
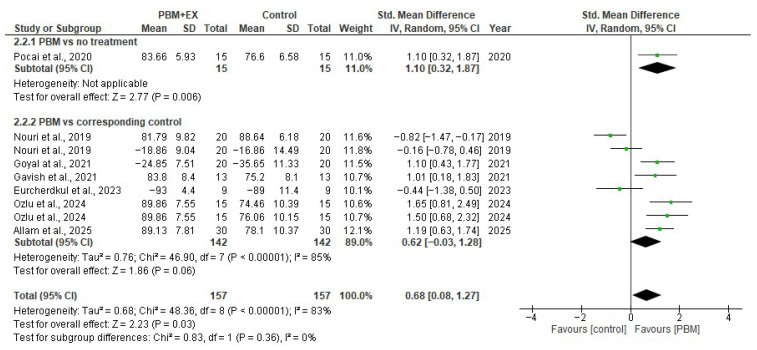
Forest plot of effectiveness of PBM on function [5,23,24,46,47,48,49].

A subgroup analysis of studies where PBM was delivered alongside exercise produced an effect in the same direction (SMD = 0.62; 95% CI –0.03 to 1.28; I^2^ = 85%), although the confidence interval crossed the null effect, indicating uncertainty regarding the magnitude of benefit in this subgroup. A single study evaluating PBM alone versus no treatment demonstrated a significant improvement in function (SMD = 1.10; 95% CI 0.32 to 1.87; *p* = 0.006). Overall, although substantial heterogeneity exists, the pooled evidence suggests that PBM is associated with improved functional outcomes in individuals with PFPS. Consistency in the direction of effects across studies supports a beneficial role for PBM, but variation in treatment protocols limits the precision of the pooled estimate (Figure 4).

## 4. Discussion

The results of this review showed that PBM, delivered in combination with exercise, provides therapeutic benefits for individuals with PFPS. Across eight RCT, PBM has significant reduction in pain intensity. Functional outcomes showed a similarly favorable pattern: five trials using the AKPS scale and three studies evaluating WOMAC scale revealed improvement in knee function with minimal heterogeneity, although substantial variability among studies contributed to high heterogeneity. These findings suggest that PBM is an effective adjunct intervention that reduces pain and enhances knee function in patients with PFPS, with the strongest evidence emerging when PBM is combined with exercise.

The findings of the present review align with and extend previous work examining the effects of PBM on musculoskeletal disorders. Prior systematic reviews have reported that PBM produces analgesic and functional benefits in knee osteoarthritis, tendinopathies, and other chronic pain conditions through mechanisms involving mitochondrial activation and modulation of inflammatory mediators [32,51,52,53].

The present review showed that PBM, when combined with structured exercise therapy, yields consistent pain modulation and functional improvements. These results reflect the growing clinical evidence that PBM may considered as adjunctive modalities and can enhance the overall effectiveness of PFPS rehabilitation when integrated with targeted strengthening programs. Nevertheless, similar to previous reports, variability in PBM parameters and inconsistent reporting across clinical trials persist as methodological challenges, underscoring the need for standardized treatment protocols and well-designed randomized trials specific to PFPS.

The therapeutic effects of PBM are primarily mediated through its interaction with mitochondrial chromophores, particularly cytochrome-c oxidase, leading to enhanced cellular energy production and modulation of inflammatory pathways. Absorption of red and near-infrared photons increases mitochondrial membrane potential and stimulates adenosine triphosphate (ATP) synthesis, thereby improving cellular metabolism and promoting tissue repair [33,54]. Moreover, it promotes neovascularization and can increase collagen synthesis, aiding in the repair of stressed or injured periarticular tissues and potentially influencing the regenerative process [55].

The supporting literature showed that PBM may have an analgesic effect as PBM energy can directly target nerve endings and slow the transmission of pain signals and increase the production of endorphins and other morphine-like substances, offering a natural pain-relieving effect [5]. Others explain that improvement in pain and function is due to the anti-inflammatory and anti-edema effects of PBM. PBM can downregulate inflammatory cytokines, which are key contributors to the pain and tissue damage associated with PFPS [53]. Also, it helps to reduce edema (swelling) and inflammation, promoting a better healing environment [56].

Although three studies demonstrated a low risk of bias [23,46,47], supported by appropriate randomization procedures, adequate allocation concealment, and the use of credible sham devices to maintain participant blinding, many trials exhibited important methodological limitations. Five studies were rated as having either multiple domains with “some concerns” or at least one domain judged as high-risk, resulting in an overall high risk of bias classification [5,23,46,47]. The most frequent sources of bias were the inadequate reporting or implementation of allocation concealment, the inability to blind treating therapists, and insufficient clarity regarding pre-specified statistical analyses. These issues may have introduced performance and reporting biases that could have influenced treatment effects. Nevertheless, missing outcome data was generally well managed across trials, with minimal loss to follow-up, reducing concerns about attrition bias. Overall, the predominance of trials with methodological shortcomings likely contributed to the observed heterogeneity and reduced confidence in the certainty of the pooled estimates.

The GRADE assessment indicates that the overall certainty of evidence supporting the effects of PBM on both pain and function in PFPS is very low, despite statistically significant pooled estimates. This downgrade was driven primarily by methodological limitations, inconsistency, and imprecision across the included studies. Most trials had issues related to unclear allocation concealment, inadequate blinding procedures, or high overall risk of bias ratings, which increases the likelihood that observed effects may be influenced by study design weaknesses rather than true treatment efficacy.

Substantial heterogeneity was present for both pain and functional outcomes (I^2^ > 75%), reflecting considerable variability in PBM parameters, co-interventions, outcome measures, and comparator conditions. Such inconsistency reduces confidence in the reproducibility of the findings, as treatment effects varied in magnitude across studies. Imprecision further contributed to the low certainty of evidence. For both pain and function, the pooled confidence intervals crossed the established minimal important difference thresholds, indicating uncertainty about whether the observed improvements translate into clinically meaningful benefits for all patients. Small sample sizes in several trials compounded this imprecision, limiting the robustness of the overall estimates.

Collectively, these factors caution against drawing firm conclusions about the magnitude of PBM’s clinical effectiveness. While the direction of effect consistently favored PBM for both pain reduction and functional improvement, the underlying evidence remains fragile. Larger, rigorously conducted randomized controlled trials with standardized PBM protocols and adequately larger samples are required to strengthen confidence in these findings and clarify the true clinical value of PBM for PFPS.

The findings of this review have important clinical implications for the management of PFPS. Across trials, PBM was typically delivered in conjunction with standard exercise-based rehabilitation, and because exercise was included in both intervention and control groups, the observed benefits primarily reflect the additional effect of PBM rather than a synergistic interaction with exercise. The reductions in pain and improvements in function suggest that PBM may help patients tolerate rehabilitation more comfortably, potentially facilitating engagement in strengthening and neuromuscular training programs that are central to PFPS management.

However, the considerable variability in PBM parameters across studies underscores the need for clinicians to use evidence-informed dosing, particularly within wavelength and energy ranges shown to be effective for musculoskeletal conditions. Although PBM alone demonstrated some short-term analgesic benefit in the few trials where it was evaluated independently, the current evidence base predominantly reflects PBM used alongside routine exercise therapy, supporting its role as an adjunct rather than a replacement for active rehabilitation. Until more standardized and methodologically robust RCTs become available, clinicians should interpret existing dosage recommendations cautiously and tailor PBM application to individual patient needs.

This review has several notable strengths. It is the first systematic review and meta-analysis to synthesize RCT, evaluating the effects of PBM specifically in patients with PFPS. A comprehensive and methodologically robust search strategy was employed across multiple databases to ensure broad coverage of the available evidence. Data extraction, risk-of-bias assessment, and GRADE were performed independently by multiple reviewers, enhancing methodological rigor and reducing the likelihood of reviewer bias. Additionally, PBM parameters were extracted in detail, providing valuable insights into treatment characteristics that may inform clinical decision-making and future trial design.

Nevertheless, the review has limitations that should be acknowledged. The overall certainty of evidence was reduced due to methodological concerns within the included studies, such as unclear allocation concealment, insufficient blinding, and variability in the reporting of PBM dosing parameters. Considerable heterogeneity was present in the pooled pain analysis, likely reflecting differences in PBM devices, wavelengths, energy densities, treatment durations, and comparator interventions. Functional outcomes, although consistent across studies, were limited by small sample sizes and imprecision relative to minimally important difference thresholds.

Although the gray literature sources were included to minimize publication bias, this approach may have resulted in the inclusion of studies that had not undergone peer review, potentially affecting the overall reliability of the evidence base. Additionally, several subgroup analyses were based on a very limited number of studies, sometimes only a single RCT, thereby restricting the robustness of these subgroup findings. Results drawn from such small subgroup samples should be interpreted with caution, as they may not accurately reflect true subgroup effects and are more susceptible to random error. Furthermore, the inclusion of only English-language studies may introduce language bias, and the small number of eligible RCTs restricts the ability to conduct more precise subgroup or dose–response analyses. These limitations highlight the need for larger, well-designed, and standardized clinical trials to strengthen the evidence base for PBM in PFPS.

## 5. Conclusions

This systematic review and meta-analysis indicate that photobiomodulation therapy (PBM) provides additional benefits for pain reduction and functional improvement in individuals with PFPS when delivered alongside the exercise-based rehabilitation that was common to both intervention and control groups. Although the direction of effect consistently favors PBM, the magnitude of improvement varies across studies, likely due to methodological limitations, heterogeneity in PBM parameters, and imprecision in effect estimates. Given these uncertainties and the very low certainty of evidence, PBM should be considered a potential adjunct within multimodal rehabilitation rather than a standalone or synergistic intervention. Further high-quality randomized controlled trials with standardized PBM protocols are needed to clarify optimal dosing parameters and to confirm the clinical relevance and durability of treatment effects.

## Figures and Tables

**Table 1:** Summary of the included studies’ characteristics and findings.

Authors	Population (Disease)	Study Design	Sample Size Male/Female	Mean Age ± SD	Measured Outcomes	Interventions Main/Comparators	Dropouts and Adverse Effects (AEs)
**Nouri et al., 2019** [24]	PFPS, (15–40) yrs, ≥2 positive PFPS tests	Single-blind RCT	40 (12 M/28 F)	31.43 ± 6.72/35.29 ± 3.27	Pain: VAS (0–10) Function: Kujala Score Pain/Function/Stiffness: WOMAC subscales and total.	G1: HILT + EX G2: Sham laser + EX	Dropouts: None reported. No adverse events reported.
**Pocai et al., 2020** [49]	PFPS, young females	RCT	30 women (15 per group).	21.87 ± 2.74 years.	Pain: (VAS) Questionnaires: KOOS-PF, AKPS, and TSK.	G1: PBM G2: No intervention	Dropouts: None reported. No adverse events reported.
**Gavish et al., 2021** [23]	Anterior knee pain (PFPS-type) in soldiers	double-blind RCT	26 15 M/11 F.	PBM = 22 ± 6 years. Sham = 23 ± 10 years.	Pain: (VAS) Kujala Score: AKPS	G1: PT + PBM group: physiotherapy EX.+ Laser G2: Sham group: PT + inactive light probes.	Dropouts: None reported. No adverse events reported.
**Goyal at al., 2021** [48]	Bilateral patellofemoral OA (KL I–II)	Randomized split-body trial	40 knees (20 knees per group).	45–60 years	Pain: (VAS) WOMAC:(pain, stiffness, physical function)	G1: LLLT + EX G2: Mobilization + heat + EX	Dropouts: 2 participants lost in follow-up No adverse events reported.
**Qayyum et al., 2022** [50]	PFPS, >3 months pain	Single-blind RCT	66 (35 M/31 F)	27.7 ± 6.7	Pain: VAS	G1: HPLT + PT G2: PT alone.	Dropouts: None No adverse events reported.
**Eurcherdkul et al., 2023** [47]	PFPS (20–50 yrs)	Double-blind RCT	18 (5 M/13 F)	36.4 ± 7.5/34.3 ± 8.8	Pain: VAS, Function: Kujala AKPS Quality of Life: SF-36v2	G1: HILT + EX G2: Sham + EX	Dropouts: None Adverse effects: None reported.
**Ozlu et al., 2024** [5]	PFPS (25–45 yrs)	RCT, single-blind (3 groups)	45 (15/group) 21 M/24 F.	33 ± 6	VAS, ROM, Strength, Kujala, LEFS, TUG	G1: HILT + EX G2: US-TENS + EX G3: US-IFC + EX	Dropouts: None No adverse events reported.
**Allam et al., 2025** [46]	PFPS (18–25 yrs)	RCT, double-blind (sham-controlled)	60 (19 M/41 F)	21.1 ± 1.8	VAS, Kujala AKPS ROM	G1: Laser acupuncture + EX G2: Sham + EX	Dropouts: None No serious adverse effects.

Abbreviation: PFPS: Patellofemoral Pain Syndrome, RCT: randomized controlled trial, PBM: photobiomodulation, EX: exercises, VAS: Visual Analog Scale, AKPS: Anterior Knee Pain Scale, TSK: Tampa Scale for Kinesiophobia, HILT: high-intensity laser therapy, LEFS: Lower-Extremity Functional Scale, TUG: Timed Up and Go, ROM: range of motion, WOMAC: Western Ontario and McMaster Universities Osteoarthritis Index, US: Ultrasound, IFC: Interferential current, TENS: Transcutaneous Electrical Nerve Stimulation, OA: Osteoarthritis.

**Table 2:** Summary of photobiomodulation parameters in included studies.

	Laser Type (LLLT/HILT/LED/Cluster)	Wavelength (nm)	Power (mW)	Spot Size (cm^2^)	Pulse Frequency (Hz)	Energy Density (J/cm^2^)	Total Energy (Joule)	Treatment Time Session	Sessions Per Week	Total Sessions	Application Site
**Nouri et al., 2019** [24]	HPLT (Nd: YAG) BTL-6000^®^	1064 nm	10 W	0.8 cm^2^	Pulsed 25% duty	120 J/cm^2^ per session	300 J per session	2 min	3/week	5 sessions	Circular movements around patellar margins (knee extended).
**Pocai et al., 2020** [49]	PBM (cluster Laser + LED) Fluence HTM^®^	Laser: 830 nm (infrared) LED: 590 nm (visible)	1650 mW	**NR**	CW	8.4 J per session,	100 (total)	2 min 1 min × 2 areas	3/week	12 sessions	Medial and lateral patellar regions (direct skin contact).
**Gavish et al., 2021** [23]	Multisource PBM (LED + Laser) **NR**	LED: 660 nm and 850 nm. laser cluster: 810 nm	LED = 50 mW/cm^2^ Laser = 200 mW	LED = 1.0 cm^2^. Laser pointer = 0.042 cm^2^. Laser = 0.033 cm^2^	LED cluster pulsed 2.5 Hz. laser beams continuous.	LED: 3 J/cm^2^ Laser pointer: 142.5 J/cm^2^. Laser:180 J/cm^2^	100–150/knee	1–2 min	2/week	8 sessions	(A) Popliteal and inguinal lymph nodes (LED cluster). (B) Around patella and max pain areas (LED cluster); (C) 3–6 muscle trigger points (single laser); (D) Lumbar L2–L5 nerve roots (laser cluster).
**Goyal at al., 2021** [48]	(LLLT), Class IIIb device **NR**	810 nm	50 mW	1 cm^2^	300 Hz	6 J/cm^2^	6 J	2 min	4/week	16 sessions	Right knee (anterior surface around patella).
**Qayyum et al., 2022** [50]	HPLT (Nd: YAG) **NR**	1064 nm	10 W	0.8 cm^2^	Pulsed mode	120 J/cm^2^	96 J per session	2 min	3/week	8 sessions	Circular movement over patellar (knee extended).
**Eurcherdkul et al., 2023** [47]	HILT Hilterapia^®^ HIRO TT	1064 nm (Nd: YAG)	10.5 W peak power 3 kW.	NR	20 Hz	1.530 J/cm^2^	3000 J per session	NR	2–3/week for 3 weeks	6 sessions	5 points surrounding the anterior knee (knee flexed at 30°).
**Ozlu et al., 2024** [5]	HILT (Nd: YAG) **NR**	1064 nm	Analgesia = 10 W Biostimulation = 7 W	25 cm^2^	25 Hz	Analgesia: 12 J/cm^2^ biostimulation: 150 J/cm^2^	4050 J	10 min	5 sessions per week.	10 sessions	Around the patella and over the quadriceps tendon and patellar tendon region.
**Allam et al., 2025** [46]	LLLT (laser acupuncture) **NR**	905 nm	100 mW	NR	NR	4 J/point	24/session	8 min	2/week for 4 weeks	8 sessions	Six knee acupuncture points: ST34, ST35, GB34, EX-LE4, SP9, SP10.

Abbreviation: LLLT: low-level laser therapy, HILT: high-intensity laser therapy, nm: nanometer, HPLT: High-power laser therapy, LED: Light-Emitting Diode, Nd: YAG: Neodymium-doped Yttrium Aluminum Garnet, mW: milliwatt, cm^2^: centimeter square, Hz: Hertz, J/cm^2^: joule per square centimeter, HPLT: high-power laser therapy, NR: not reported.

**Table 3:** Quality of evidence (GRADE).

Outcome Measured	N. of Part. (Studies)	Study Limitation	Inconsistency	Indirectness	Imprecision	Publication bias	Overall Quality of Evidence	Effect Estimate SMD [95% CI]	Effect Size	Direction
**Pain**	340 (8)	Serious ^a^	Serious **^b^**	Not Serious	Serious ^d^	Possible ^e^	Very Low Ꚛꓳꓳꓳ	−0.83 [−1.40, −0.27]	Large	Favor to PBM
**Function**	314 (7)	Serious ^a^	Serious **^b^**	Not Serious	Serious ^d^	Possible ^e^	Very Low Ꚛꓳꓳꓳ	0.68 [0.08, 1.27]	Medium	Favor to PBM

GRADE, Grading of Recommendations Assessment, Development and Evaluation; SMD, Standardize Mean Difference; CI, Confidence Interval. ^a^ Study limitation: Allocation concealment was not clearly reported and lack of blinding of participants or assessors and therapists. ^b^ Inconsistency: Significant heterogeneity in meta-analysis, I^2^ > 75; ^d^ Imprecision: Small sample size with wide confidence interval, less than 400 participants, pooled effect crossed the minimally important difference (MID). ^e^ Publication bias: Funnel plots were underpowered (<10 studies). Potential small-study effects were considered but not formally downgraded. Ꚛꓳꓳꓳ: Very low quality of evidence: the true effect is probably markedly different from the estimated effect with very little confidence to the effect estimate.

## Data Availability

Data is contained within the article. Data was presented in excel sheet and handled by the corresponding author. Data is available upon request by the editor. The summary of this data was presented in this study and included in the article/Supplementary Material. Further inquiries can be directed to the corresponding author.

## Supplementary Materials

The following supporting information can be downloaded at: https://www.mdpi.com/article/10.3390/jcm15010020/s1, Table S1: PRISMA 2020 Checklist; Table S2: History and Search Details for PubMed and Medline Ultimate databases.

## Abbreviations

The following abbreviations are used in this manuscript:

AKPS	Kujala Anterior Knee Pain
ATP	Adenosine triphosphate
GRADE	Grading of Recommendations Assessment, Development and Evaluation
HILT	High-intensity laser therapy
HPLT	High-power laser therapy
LLLT	Low-level laser therapy
NPRS	Numeric Pain Rating Scale
PBM	Photobiomodulation
PEDro	Physiotherapy Evidence Database
PFPS	Patellofemoral Pain Syndrome
PICO	Population, Intervention, Comparison, and Outcome
PRISMA	Preferred Reporting Items for Systematic Review and Meta-Analysis
PROSPERO	International Prospective Register of Systematic Reviews
RCT	Randomized controlled trials
RoB 2	Cochrane Risk of Bias 2.0
SMD	Standardize Mean difference
VAS	Visual Analog Scale
WOMAC	Western Ontario and McMaster Universities Osteoarthritis Index
