# Supplementary material for: The Effectiveness of Photobiomodulation Therapy on Pain and Function in Patients with Patellofemoral Pain Syndrome—A Systematic Review and Meta-Analysis
Source: J Clin Med. 2025 Dec 19;15(1):20. doi: 10.3390/jcm15010020 (PMC12786645; doi:10.3390/jcm15010020)
Supplement: Supplementary file 1 [file jcm-15-00020-s001.zip › Table S2.pdf]

History and Search Details for **PubMed Advanced Search Builder**

#16	#5 add with AND #15<br>Search: **(((((((((photobiomodulation therapy[Title/Abstract]) OR (photobiomodulation therapy[Title/Abstract])) OR (low-level laser therapy[Title/Abstract])) OR (high-intensity laser therapy[Title/Abstract])) OR (laser therapy[Title/Abstract])) OR (light therapy[Title/Abstract])) OR (PBM[Title/Abstract])) OR (HPLT[Title/Abstract])) OR (LLLT[Title/Abstract])) OR (HILT[Title/Abstract])) AND ((((patellofemoral pain syndrome[Title/Abstract]) OR (anterior knee pain[Title/Abstract])) OR (chondromalacia patellae[Title/Abstract])) OR (patellofemoral dysfunction[Title/Abstract]))**<br>("photobiomodulation therapy"[Title/Abstract] OR "photobiomodulation therapy"[Title/Abstract] OR "low level laser therapy"[Title/Abstract] OR "high intensity laser therapy"[Title/Abstract] OR "laser therapy"[Title/Abstract] OR "light therapy"[Title/Abstract] OR "PBM"[Title/Abstract] OR "HPLT"[Title/Abstract] OR "LLLT"[Title/Abstract] OR "HILT"[Title/Abstract]) AND ("patellofemoral pain syndrome"[Title/Abstract] OR "anterior knee pain"[Title/Abstract] OR "chondromalacia patellae"[Title/Abstract] OR "patellofemoral dysfunction"[Title/Abstract])	9
#15	Search: **(((((((((photobiomodulation therapy[Title/Abstract]) OR (photobiomodulation therapy[Title/Abstract])) OR (low-level laser therapy[Title/Abstract])) OR (high-intensity laser therapy[Title/Abstract])) OR (laser therapy[Title/Abstract])) OR (light therapy[Title/Abstract])) OR (PBM[Title/Abstract])) OR (HPLT[Title/Abstract])) OR (LLLT[Title/Abstract])) OR (HILT[Title/Abstract])**<br>"photobiomodulation therapy"[Title/Abstract] OR "photobiomodulation therapy"[Title/Abstract] OR "low level laser therapy"[Title/Abstract] OR "high intensity laser therapy"[Title/Abstract] OR "laser therapy"[Title/Abstract] OR "light therapy"[Title/Abstract] OR "PBM"[Title/Abstract] OR "HPLT"[Title/Abstract] OR "LLLT"[Title/Abstract] OR "HILT"[Title/Abstract]	21258
#14	Search: **HILT[Title/Abstract]**<br>"HILT"[Title/Abstract]	206
#13	Search: **LLLT[Title/Abstract]**<br>"LLLT"[Title/Abstract]	2704
#12	Search: **HPLT[Title/Abstract]**<br>"HPLT"[Title/Abstract]	37
#11	Search: **PBM[Title/Abstract]**<br>"PBM"[Title/Abstract]	4669
#10	Search: **light therapy[Title/Abstract]**<br>"light therapy"[Title/Abstract]	3617
#9	Search: **laser therapy[Title/Abstract]**<br>"laser therapy"[Title/Abstract]	13130
#8	Search: **high-intensity laser therapy[Title/Abstract]**<br>"high intensity laser therapy"[Title/Abstract]	206
#7	Search: **low-level laser therapy[Title/Abstract]**<br>"low level laser therapy"[Title/Abstract]	3623

#6	Search: **photobiomodulation therapy[Title/Abstract]**<br>"photobiomodulation therapy"[Title/Abstract]	1320
#5	Search: **(((patellofemoral pain syndrome[Title/Abstract]) OR (anterior knee pain[Title/Abstract])) OR (chondromalacia patellae[Title/Abstract])) OR (patellofemoral dysfunction[Title/Abstract])**<br>"patellofemoral pain syndrome"[Title/Abstract] OR "anterior knee pain"[Title/Abstract] OR "chondromalacia patellae"[Title/Abstract] OR "patellofemoral dysfunction"[Title/Abstract]	3750
#4	Search: **patellofemoral dysfunction[Title/Abstract]**<br>"patellofemoral dysfunction"[Title/Abstract]	61
#3	Search: **chondromalacia patellae[Title/Abstract]**<br>"chondromalacia patellae"[Title/Abstract]	310
#2	Search: **anterior knee pain[Title/Abstract]**<br>"anterior knee pain"[Title/Abstract]	2670
#1	Search: **patellofemoral pain syndrome[Title/Abstract]**<br>"patellofemoral pain syndrome"[Title/Abstract]	975

Medline Ultimate database

#3	#1 AND #2	5
#2	(photobiomodulation therapy or low level laser therapy or photobiomodulation or lllt) AND (rct or randomized control trial or randomized controlled trial)	925
#1	patellofemoral pain syndrome or patellofemoral or patellofemoral syndrome or anterior knee pain syndrome AND Randomized Controlled trial	4004
